# NRSN2 promotes the malignant behavior of HPV-transfected laryngeal carcinoma cells through AMPK/ULK1 pathway mediated autophagy activation

**DOI:** 10.1080/15384047.2024.2334463

**Published:** 2024-04-03

**Authors:** Fan Guo, Wulin Wen, Zhipeng Mi, Chao Long, Qiangyou Shi, Meihua Yang, Jia Zhao, Ruixia Ma

**Affiliations:** a School of Clinical Medicine, Ningxia Medical University, Yinchuan, Ningxia, P.R. China; b Otolaryngology Department, The First People’s Hospital of Yinchuan, Otolaryngology Head and Neck Surgery Hospital, Yinchuan, Ningxia, P.R. China; c The Second Clinical Medical College, Ningxia Medical University, Yinchuan, Ningxia, P.R. China; d Department of Otolaryngology Head and Neck Surgery, Gansu Maternal and Child Health Care Hospital, Lanzhou, Gansu, P.R. China

**Keywords:** Laryngeal carcinoma, autophagy, HPV, NRSN2, AMPK/ULK1 pathway

## Abstract

Neurensin-2 (NRSN2) performs a pro-carcinogenic function in multiple cancers. However, the function of NRSN2 in HPV-infected laryngeal carcinoma (LC) remains unclear. HPV transfection was performed in LC cells. The mRNA and protein levels were monitored using RT-qPCR, immunoblotting, and IF. Cell viability and proliferation were found using the CCK-8 assay and Edu staining. Cell invasion, migration, and apoptosis were probed using the Transwell, wound healing, and flow cytometry, respectively. The autophagosome was observed using TEM. NRSN2 was overexpressed in HPV-transfected LC cells. Inhibition of NRSN2 restrained the autophagy and malignant behavior of HPV-transfected LC cells. Meanwhile, the inhibition of AMPK/ULK1 pathway limited the increased autophagy of HPV-transfected LC cells caused by NRSN2 overexpression. Furthermore, NRSN2 knockdown inhibits autophagy by suppressing AMPK/ULK1 pathway, thereby restraining the malignant behavior of HPV-transfected LC cells. Our research confirmed that HPV transfection increased the autophagy and malignant behavior of LC cells by regulating the NRSN2-mediated activation of the AMPK/ULK1 pathway, offering a new target for cure of LC.

## Introduction

Head and neck cancer (HNC), with heterogeneity and high recurrence rate, is a malignant tumor occurring in the mucous epithelium of the upper respiratory tract.^1^ Laryngeal carcinoma (LC) is one of the most common cancers of HNC, accounting for approximately one-third of total HNC.^2,3^ In recent years, the incidence of LC has continued to rise, with a 5-year overall survival rate of only 50%, and many patients are already in the advanced stage when discovered.^4,5^ The treatment methods for LC include total laryngectomy, laser transoral throat microsurgery, radiotherapy, and chemotherapy, among which surgical resection is the most effective method.^6,7^ However, due to the crucial role of the larynx in phonation, surgical resection may have a significant impact on the patient’s life.^8^ Therefore, exploring potential diagnostic and therapeutic targets for LC is urgent. In recent years, more and more studies have found that among the many pathogenic factors of LC (smoking, drinking, air pollution, human papillomavirus (HPV) infection, etc.), HPV (especially HPV16) infection may be one of the most important factors.^9,10^ It is reported that the HPV infection rate of LC is high, and HPV infection has a positive effect on the development of LC.^9,11^ However, how HPV participates in the development of LC is still unknown.

Autophagy, a cellular degradation pathway, performs a vital function in sustaining cell homeostasis.^12^ There is evidence that in many cases, the autophagy of cancer cell supports tumor growth.^13,14^ Lin C et al.^15^ covered that inhibition of autophagy via blocking the AMPK/ULK1 pathway can restrain the development of prostate cancer. Autophagy inhibition has become a promising therapeutic target for LC.^16^ The study of Guo Y et al.^17^ covered that the inhibition of autophagy enhances the chemosensitivity of LC cells to cisplatin. Chen XH et al.^18^ found that autophagy inhibition reduces the propagation and migration of LC stem cells. The research of Li G et al.^19^ proved that miR-339-5p reduces the resistance of LC to cisplatin by inhibiting autophagy. In addition, studies have found that compared with clinically normal laryngeal tissues, the level of autophagy marker LC3B and the number of autophagosomes in HPV-transfected respiratory papilloma tissues are significantly higher.^20^ This suggests that HPV may regulate laryngeal cancer progression by influencing autophagy.

Neurensin-2 (NRSN2), a protein localized on the cell membranes, has been reported to perform a cancer-promoting role in a variety of tumors in recent years.^21^ For example, studies have found that high NRSN2 expression is associated with the malignant phenotype of ovarian cancer.^22^ The report of Keremu A et al.^23^ revealed that NRSN2 promotes the growth of osteosarcoma cells. Additionally, a research has displayed that NRSN2 accelerates the development of esophageal squamous cell carcinoma.^24^ Nevertheless, the action of NRSN2 in LC is dim.

Hence, we hypothesized that NRSN2 expression is up-regulated and autophagy is enhanced in HPV-transfected LC cells, thus promoting the growth of HPV-transfected LC cells and inhibiting the apoptosis of HPV- transfected LC cells. We will establish a stable HPV-transfected LC cells and perform knockdown or overexpression of NRSN2 on these cells to explore the role of NRSN2 in HPV-mediated malignant behavior in LC, giving the diagnosis and treatment of LC a novel underlying target.

## Methods

### Cell culture and treatment

The LC cells TU212 and TU138 were cultivated in RPMI 1640 (Gibco) appended with 10% fetal bovine serum (FBS, Gibco), penicillin/streptomycin (Gibco), and humidity of 95% air and 5% CO_2_ at 37°C. For the inhibition of ULK1 and AMPK, the SBI-0206965 (SBI, 10 μM, MCE, China) and Compound C (Com, 10 μM, MCE) were applied to the cells for 24 h, respectively. Rapamycin (RAP, 20 μM, MCE) was added to activate the autophagy.

### Generation of cells stably expressing HPV16E7

TU212 and TU138 cells were planted in the petri dish of 6 cm at a denseness of 1.5 × 10^6^ cells and cultivated to about 90% denseness. Then, the recombinant vector pLXSN16E7 (Addgene, USA) containing the HPV16E7 genome was stably transfected into TU212 and TU138 cells via the Lipofectamine 3000 (Invitrogen, USA). 24 h later, the medium was appended with G418 (500 μg/mL, Invitrogen) to screen the cells stably expressing HPV16E7 (TU212/HPV and TU138/HPV) for 8 consecutive weeks. RT-qPCR and immunoblotting were applied to assay if the cells stably expressing HPV16E7 were successful or not.

### Cell transfection

For the overexpression of NRSN2, NRSN2 overexpression vectors (ov-NRSN2) and the matching negative vectors (ov-NC) were constructed via Sangon Biotech Co., Ltd (China). For the NRSN2 knockdown, NRSN2 (sh-NRSN2, target sequence: 5’-AGGGTGTACAGCCACTATTTA-3’) knockdown vectors and the matching negative vector (sh-NC) were constructed via Sangon Biotech Co., Ltd. These vectors were transfected into TU212/HPV and TU138/HPV cells via the Lipofectamine 3000 (Invitrogen).

### Transmission electron microscopy (TEM) to detect the autophagosomes

The cells of each group were prefixed in 2.5% glutaraldehyde for 2 h and then cleaned 3 times in 0.1 M phosphate buffer (PB) for 60 s each time. Fixed at 4°C for another 1 h in a 1% osmic acid. Then, it was washed with 0.1 M PB for 3 times, stained in 1% uranyl acetate under 25°C for 1 h, and cleaned in ddH_2_O for 3 times. The cells were progressively dehydrated in varying concentrations of ethanol solutions for 8 min, followed by cleaning in propylene oxide for 10 min. Later, the cells were coated with a compound of EPON812 resin and propylene oxide (2:1) under 4°C for 4 h, and then in pure EPON812 resin overnight. After roasting at 60°C for 24 h, the samples were cut into ultra-thin slices (70-nm thick) using the Ultracut UCT Microtome (Leica Biosystems, USA) and dyed in 1% uranium acetate for 20 min and lead citrate for 5 min. A Tecnai Spirit 120 kV TEM (FEI, USA) was applied to obtain the images.

### Immunofluorescence (IF) staining

Cells of each group were inoculated in petri dishes with coverslip to prepare cell slides, which were fixed in 4% paraformaldehyde for 30 min, cleaned with PB saline (PBS) for 3 times, and penetrated in 0.2% Triton X-100 for 3 min. Then, the cell slide was cleaned with PBS for 3 times, and blocked with goat serum at 25°C for 30 min. The anti-LC3B (ab192890, 1:200, Abcam, UK) was appended and placed at 4°C for 12 h and then the cell slide was cleaned with PBS for 3 times. The cell slide was further mixed with the secondary antibody (ab150083, 1:1000, Abcam) in the dark for 2 h. After cleaned in PBS for 3 times, the cell slide was dyed with DAPI, and then photographed under an AX-70 fluorescent microscopy (Leica Microsystems Inc.).

### Reverse transcription‑quantitative polymerase chain reaction (RT‑qPCR)

Using the Trizol (Invitrogen, USA), the overall RNA from the above treated cells was isolated. The SuperScript™ VILO™ cDNA Synthesis Kit (Thermo Fisher Scientific, USA) was applied to reverse the overall RNA into cDNA according to the instructions. Subsequently, the mRNA level of HPV16E7 and NRSN2 were probed via qPCR with SYBR Green PCR Master Mix (Takara, China) on the 7900HT Fast Real-Time PCR System (Applied Biosystems, USA). The reference gene for normalization was GADPH. The shift of the mRNA level of HPV16E7 and NRSN2 was quantified via 2^−∆∆Ct^ method. The Forward (F) and Reverse (R) primer sequences of HPV16E7, NRSN2, and GAPDH were offered: NRSN2-F: 5’-GATGGCAAGTGGTATGGGGTC-3’, NRSN2-R: 5’-CGAGGACAGGCTGATCTTCC-3’; GAPDH-F: 5’-GGAGCGAGATCCCTCCAAAAT3’, GAPDH-R: 5’-GGCTGTTGTCATACTTCTCATGG-3’.

### Immunoblotting

The RIPA lysate (Beyotime, China) was used to isolate the overall protein from the above treated cells and the concentration of overall protein was probed via BCA kit (Beyotime, China). 24 μg of overall protein was isolated via SDS-PAGE and electrotransferred onto PVDF membrane (Millipore, USA) at 4°C. Afterward, 5% skim milk was applied to incubate the PVDF membrane placed in at 25°C for 1 h. The primary antibodies were then cultivated with the PVDF membrane all-night at 4°C. The membrane was placed in specific secondary antibody for a further 2 h at 25°C. Finally, the protein bands were tested via the enhanced chemiluminescence (ECL) system (Thermo Fisher Scientific) and analyzed using Image J. The above antibodies including anti-HPV16E7 (ab308180, 1:1000), anti-NRSN2 (ab237739, 1:5000), anti-LC3B (ab192890, 1:2000), anti-p-ULK1(S555) (#5869, 1:1000), anti-p62 (ab109012, 1:4000), anti-p-AMPK(T172) (#2535, 1:1000), anti-β-actin (ab8227, 1:5000), and secondary antibody (ab6721, 1:10000) were acquired from Abcam and CST (USA).

### 5-ethynyl-2-deoxyuridine (EdU) staining

Using the BeyoClick™ EdU-555 Cell proliferation test kit (Beyotime, China) to confirm the cell proliferation. The cells of the different treatment groups were placed in 20 mmol/L EdU for 2 h. Then, the cells were immobilized at room temperature with 4% paraformaldehyde for 15 min. After scoured with PBS, the cells were permeated with 0.3% Triton X-100, then washed with PBS and placed in Click reaction solution at 25°C in the dark for 30 min. The cells were dyed with Hoechst 33,342 for 10 min and observed with an AX-70 fluorescent microscopy (Leica Microsystems Inc.).

### Cell counting kit-8 (CCK-8) assay

A 96-well plate was applied to seed cells from each treatment group (at a denseness of 7.5 × 10^3^ cells) and cultivated for 24 h, 48 h, and 72 h. Following this, the supernatant was dislodged, and 10 μl of CCK-8 solution was appended before being incubated for 2.5 h. Subsequently, using the SpectraMax 190 microplate reader (Molecular Devices, LLC., USA) to probe the absorbance at 450 nm.

### Wound healing assay

Cells from each treatment group (at a denseness of 2.5 × 10^5^ cells) were planted in 12-well plates and cultivated until confluent. Monolayers were scratched using a gun tip of 200 μl, washed by PBS, and then cultivated in serum-free RPMI 1640 for 24 h. The width of scratches was observed and probed by an AX-70 fluorescent microscopy (Leica Microsystems Inc.) at 0 h and 24 h, respectively.

### Transwell assay

First, add the Matrigel (BD Biosciences) to the Transwell upper chamber. 4 h later, cells of each group were planted into the upper chamber and cultured in serum-free RPMI 1640 (2.5 × 10^4^ cells). Then, the lower chamber was appended RPMI 1640 possessing 10% FBS. After 24 h cultivation, the cells in the lower side of the upper chamber were fixed with methanol for 0.5 h, and then dyed by 0.1% crystal violet (Thermo Fisher Scientific) for 1 h. Finally, the invasive cells were monitored under an AX-70 fluorescent microscopy (Leica Microsystems Inc.).

### Flow cytometry

The cells of each treatment group were gathered and scoured with icy PBS, and then re-suspended with the binding solution (Beyotime). Annexin V-FITC and propyl iodide staining solution (Beyotime) were appended and incubated at 25°C for 20 min in the dark. Then, the cells were analyzed using BD LSRFortessa X20 (USA).

### Statistical analysis

The SPSS 26.0 software (SPSS, Inc.) was applied to analyze the data, which was expressed as mean ± standard deviation (SD). The groups’ comparison and multiple groups’ comparison were analyzed by *t*-test and one-way ANOVA following Tukey’s test, respectively. All experiments were performed in triplicates at least. When the value of *p* < .05, the difference was identified as significance.

## Results

### The expression of NRSN2 was highly in HPV-transfected LC cells

**Figure 1.**
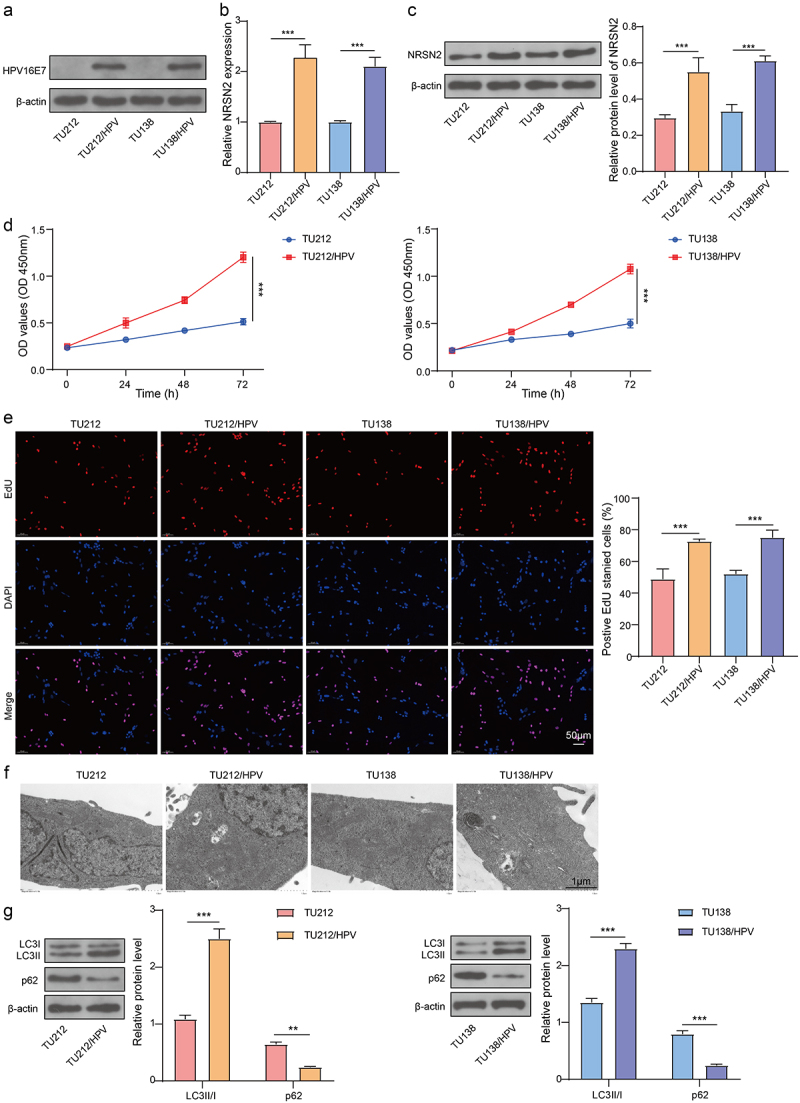
The expression of NRSN2, cell proliferation and autophagy of HPV-transfected LC cells were enhanced.

The cells stably expressing HPV16E7 (TU212/HPV and TU138/HPV) were constructed. As shown in Figures 1(a), the protein levels of HPV16E7 were highly in TU212/HPV and TU138/HPV, which indicated that the cells stably expressing HPV16E7 were successfully constructed. Then, the mRNA and protein levels of NRSN2 were assessed by RT-qPCR and immunoblotting. The consequences revealed that the HPV transfection observably increased the expression of NRSN2 (Figures 1b,c). Thus, it is indicated that HPV transfection can increase the expression of NRSN2 in LC cells.

The cells stably expressing HPV16E7 (TU212/HPV and TU138/HPV) were constructed. (a) immunoblotting was applied to assess the protein levels of HPV16E7. RT-qPCR (b), immunoblotting (c) were applied to assess the mRNA and protein levels of NRSN2. The cell viability (d), proliferation (e) were tested by CCK-8 assay and EdU staining. (F) The autophagosome was observed by TEM. (G) The protein levels of LC3B and p62 were tested using immunoblotting. **p <.01, ***p <.001.z

### The proliferation and autophagy of HPV-transfected LC cells were enhanced

We further examined the proliferation and autophagy of parental cells (TU212 and TU138) and HPV-transfected cells (TU212/HPV and TU138/HPV). The result indicated that the cell vitality and EdU positive ratio of TU212/HPV and TU138/HPV were observably enhanced than those of TU212 and TU138 (Figure 1 d,e). Furthermore, compared with TU212 and TU138, the TU212/HPV and TU138/HPV cells have more autophagosomes, as observed by TEM (Figure 1f). Moreover, the level of LC3B in TU212/HPV and TU138/HPV cells was markedly boosted than TU212 and TU138 cells, while the level of p62 in TU212/HPV and TU138/HPV cells was observably inhibited than TU212 and TU138 cells (Figure 1g). Hence, the consequences indicated that HPV transfection increased the proliferation and autophagy of LC cells.

### Inhibition of NRSN2 expression restrained the autophagy and malignant behavior of HPV-transfected LC cells

**Figure 2.**
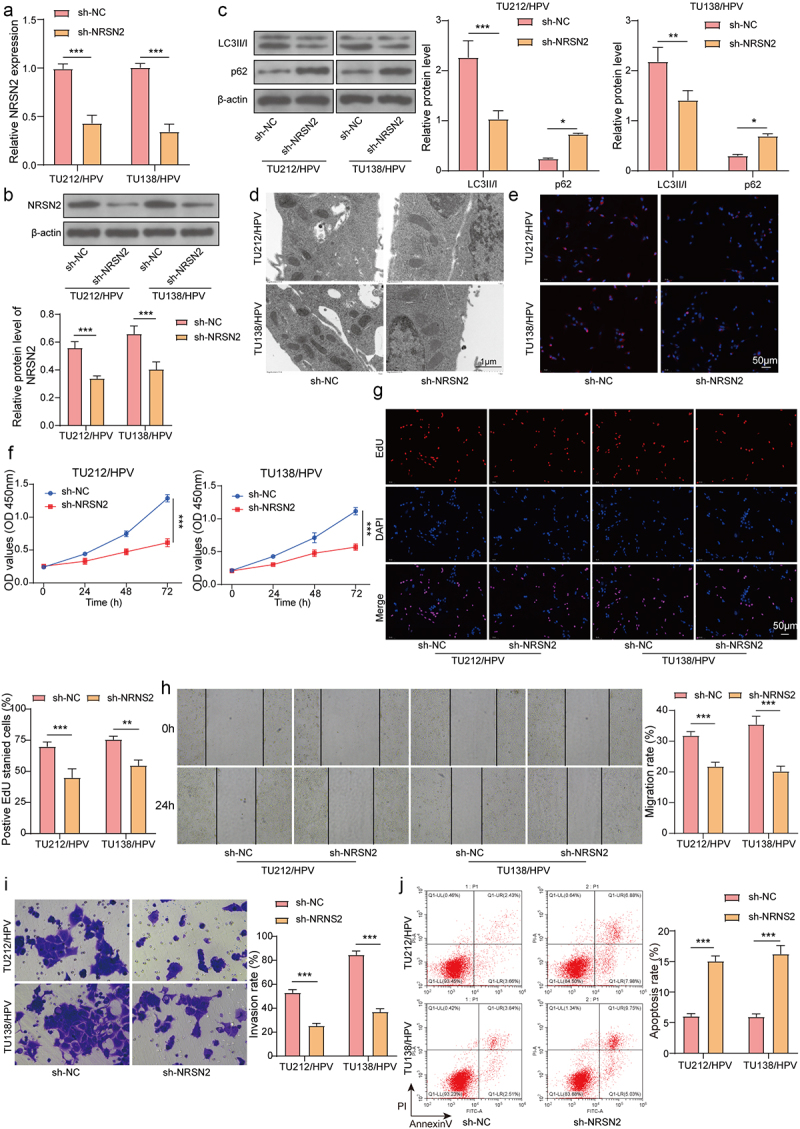
Inhibition of NRSN2 expression restrained the autophagy and malignant behavior of HPV-transfected LC cells.

To investigate the function of NRSN2 in HPV-mediated malignant behavior in LC, we knocked down NRSN2 in TU212/HPV and TU138/HPV cells. As shown in Figures 2(a,b), sh-NRSN2 transfection dramatically reduced the expression of NRSN2 compared with sh-NC transfection. Moreover, NRSN2 knockdown markedly decreased LC3B expression and increased p62 expression (Figures 2c). Similarly, the fluorescence intensity of LC3B and the number of autophagosome in TU212/HPV and TU138/HPV cells was visibly reduced by NRSN2 inhibition (Figure 2d,e). Furthermore, NRSN2 knockdown weakened the cell vitality and EdU positive ratio (Figure 2f,g). Meanwhile, the capacities of migration and invasion of TU212/HPV and TU138/HPV cells were observably inhibited via silencing NRSN2 (Figure 2h,i). Otherwise, NRSN2 knockdown aggrandized the apoptosis ratio of cells (Figure 2j). Altogether, the autophagy and malignant behavior of HPV-transfected LC cells were refrained by NRSN2 knockdown.

knocking down NRSN2 in TU212/HPV and TU138/HPV cells. RT-qPCR (a), immunoblotting (b) were applied to test the mRNA and protein levels of NRSN2. (c) The protein levels of LC3B and p62 were tested using immunoblotting. (d) The autophagosome was observed by TEM. (e) IF was applied to assess the expression of LC3B. The cell viability (f), proliferation (g), migration (h), and invasion (i) abilities were tested by CCK-8 assay, EdU staining, wound healing assay, and Transwell assay, respectively. (j) The apoptosis was assessed using flow cytometry. *p <.05, **p <.01, ***p <.001.

### Overexpression of NRSN2 upregulates autophagy via activating AMPK/ULK1 pathway

**Figure 3.**
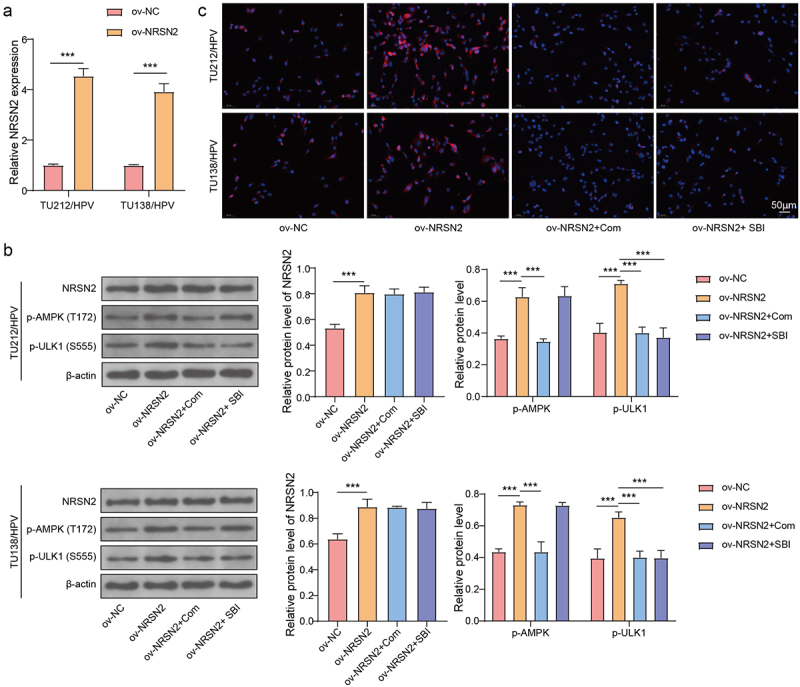
Overexpression of NRSN2 upregulates autophagy by activating AMPK/ULK1 pathway.

**Figure 4.**
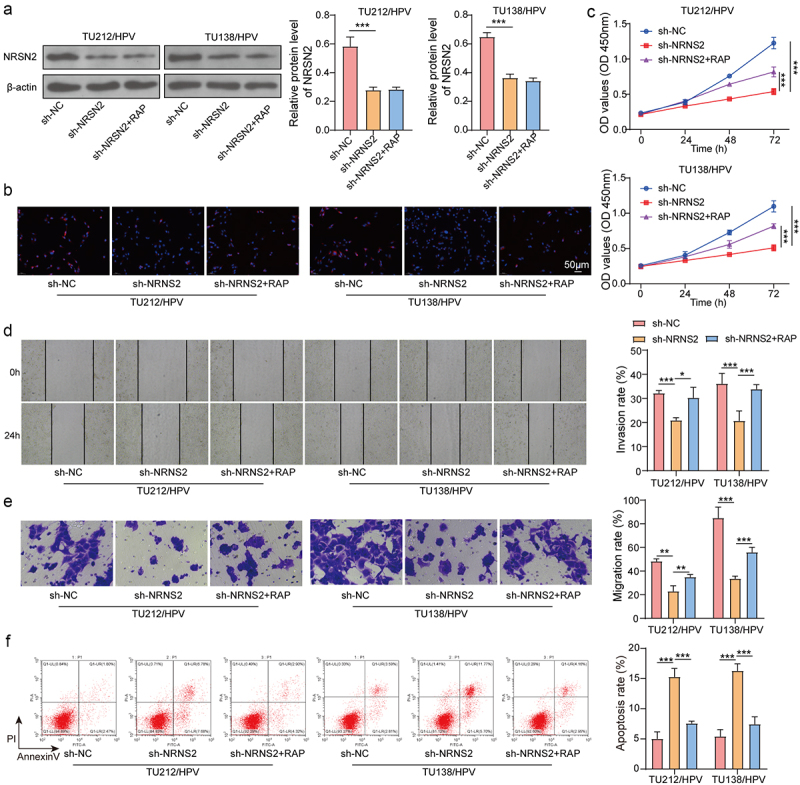
Knockdown of NRSN2 inhibits autophagy by suppressing AMPK/ULK1 pathway, thereby restraining the malignant behavior of HPV-transfected LC cells.

The AMPK/ULK1 pathway performs a vital impact in the activation of autophagy 15, 25. To test the function of AMPK/ULK1 pathway on NRSN2-mediated autophagy activation in TU212/HPV and TU138/HPV cells, we performed overexpression of NRSN2 and inhibition of AMPK/ULK1 in TU212/HPV and TU138/HPV cells. The result of Figures 3A and 3B showed that the NRSN2 overexpression was successful. In addition, we found that NRSN2 overexpression increased p-AMPK(T172) and p-ULK1(S555) levels, while Com (an inhibitor of AMPK) treatment decreased p-AMPK(T172) and p-ULK1(S555) levels (Figure 4B). Otherwise, the increased p-ULK1(S555) level caused by NRSN2 overexpression was decreased by SBI (an inhibitor of ULK1) (Figure 3B). Furthermore, the enhanced LC3B level contributed by NRSN2 overexpression was lessened by treatment of Com and SBI (Figure 3C). Broadly speaking, NRSN2 overexpression activates the autophagy by regulating AMPK/ULK1 pathway.

Overexpression of NRSN2 and inhibition of AMPK/ULK1 using SBI (an ULK1 inhibitor) and Com (an AMPK inhibitor) in TU212/HPV and TU138/HPV cells were performed. (a) The mRNA level of NRSN2 was tested using RT-qPCR. (b) The protein level of NRSN2, p-AMPK(T172), and p-ULK1(S555) were assessed using immunoblotting. (c) IF was applied to assess the expression of LC3B. ***p <.001.

Knockdown of NRSN2 and inhibition of autophagy using RAP in TU212/HPV and TU138/HPV cells were executed. (a) The protein level of NRSN2 was assessed using immunoblotting. (b) The expression of LC3B was tested using IF. The cell viability (c), migration (d), and invasion (e) abilities were tested by CCK-8 assay, wound healing assay, and Transwell assay, respectively. (f) The apoptosis was assessed using flow cytometry. *p <.05, **p <.01, ***p <.001.

### Knockdown of NRSN2 inhibits autophagy by suppressing AMPK/ULK1 pathway, thereby restraining the malignant behavior of HPV-transfected LC cells

To confirm the effect of autophagy on NRSN2-mediated malignant behavior of TU212/HPV and TU138/HPV cells, we performed inhibition of NRSN2 and autophagy in TU212/HPV and TU138/HPV cells. The outcome displayed that inhibition of NRSN2 obviously lessened the levels of NRSN2, while the autophagy activator RAP treatment had no impact on NRSN2 expression (Figure 4a). Moreover, the decreased LC3B level caused by NRSN2 inhibition was increased by treatment of RAP (Figure 4b). Otherwise, NRSN2 knockdown weakened the cell vitality, while RAP treatment restored the cell vitality (Figure 4c). The reduced migration and invasion of TU212/HPV and TU138/HPV cells caused by NRSN2 inhibition was increased by RAP treatment (Figure 4d,e). Meanwhile, NRSN2 inhibition increased the apoptosis ratio of cells, while treatment of RAP reversed these effects (Figure 4f). In summary, Knockdown NRSN2 restrains the malignant behavior of HPV-transfected LC cells by suppressing autophagy.

## Discussion

In recent years, the incidence of LC has continued to raise, with a 5-year overall survival rate of only 50%, and many patients are already in the advanced stage when discovered.^4,5^ HPV infection performs a vital function in the progression of LC ,^9–11^ Our research displayed that HPV transfection augmented the autophagy and growth of LC cells by regulating the NRSN2-mediated activation of the AMPK/ULK1 pathway.

NRSN2 has been reported to perform a cancer-promoting role in a variety of tumors in recent years.^21^ Ren F et al. reported that NRSN2 enhanced the cell proliferation and tissue growth of breast cancer.^26^ The research of Wang G et al. indicated that NRSN2 enhances the proliferation, invasion, and migration of colorectal cancer cells.^27^ Besides, a research has revealed that inhibition of NRSN2 restrains the proliferation, migration, and invasion of esophageal squamous cell carcinoma.^24^ Congruously, our research displayed that NRSN2 knockdown restrained the proliferation, migration, and invasion of HPV-transfected LC cells. Meanwhile, the apoptosis in HPV-transfected LC cells was increased via inhibition of NRSN2.

Autophagy, a cellular degradation pathway, has been reported supports tumor growth.^14^ Huang HY et al. found that autophagy inhibitors significantly increased LC cell apoptosis and death both *in vitro* and *in vivo*.^28^ The report of Chen L et al. showed that miR-101 lowered the autophagy, proliferation and enhanced the apoptosis in LC cells.^29^ In addition, studies have indicated that compared with normal laryngeal tissues, HPV-infected respiratory papilloma tissues have more LC3B expression and autophagosomes.^20^ In this study, we found that HPV transfection enhanced the autophagy of LC cells. Meanwhile, inhibition of NRSN2 lessened the proliferation, migration, and invasion and enhanced the apoptosis in HPV-transfected LC cells by reducing autophagy. AMPK/ULK1 pathway performs vital role in autophagy. The AMPK/ULK1 pathway performs a vital function in regulation of autophagy.^15,25,30^ Conformably, our investigation demonstrated that inhibition of the AMPK/ULK1 pathway can reverse the increased autophagy and cell growth and decreased apoptosis caused by overexpression of NRSN2. However, we did not probe the molecular mechanics through which NRSN2 acts on AMPK/ULK1, that is a limitation in our study. We will continue to research how NRSN2 acts on AMPK/ULK1 further.

## Conclusions

In summary, our study demonstrated that HPV transfection enhanced the autophagy and growth of LC cells by regulating the NRSN2-mediated activation of the AMPK/ULK1 pathway. Our current study provides a new potential target for the diagnosis and treatment of LC.

## Data Availability

The datasets used and/or analyzed during the current study are available from the corresponding author on reasonable request.
